# Underestimation of Low-Density Lipoprotein Cholesterol (LDL-C) Using the Friedewald Equation at Higher Triglyceride Levels: Evidence From a Method-Comparison Study

**DOI:** 10.7759/cureus.116466

**Published:** 2026-09-18

**Authors:** Divya Gupta, Abhijit P Pakhare, Tulasi Sindhuja, Rajendra K Shukla, Ashish A Jadhav, Rashmi Chowdhary, Ashok Kumar

**Affiliations:** 1 Department of Biochemistry, People's College of Medical Sciences and Research Centre, Bhopal, IND; 2 Department of Biochemistry, All India Institute of Medical Sciences (AIIMS) Bhopal, Bhopal, IND; 3 Department of Community and Family Medicine, All India Institute of Medical Sciences, Bhopal, IND; 4 Department of Biochemistry, All India Institute of Medical Sciences, Bhopal, IND

**Keywords:** atherosclerosis, cardiovascular diseases, friedewald’s equation, low-density lipoprotein cholesterol (ldl-c), triglyceride

## Abstract

Background: In biochemical laboratories, low-density lipoprotein cholesterol (LDL-C) levels are generally measured using the widely accepted Friedewald equation: $\mathrm{LDL\mathrm{-}C} = \mathrm{TC - HDL} - \mathrm{\frac{TG}{5}}$, where TC represents total cholesterol, and HDL stands for high-density lipoprotein. In the case of high serum triglycerides (TGs), the LDL-C value is underestimated using the Friedewald equation. Our study aimed to assess the reliability of calculated LDL-C (cLDL-C) compared with directly measured LDL-C (dLDL-C) in categorizing dyslipidemia patients across different TG levels.

Methods: cLDL-C was estimated using the Friedewald equation, and dLDL-C was measured by the homogeneous method. Bland-Altman plots (BAPs) were plotted to analyze the agreement between dLDL-C and cLDL-C. We used Lind’s concordance correlation coefficient to evaluate the correlation between stroke volume and cardiac output between the two methods. Mean bias with standard deviation and 95% confidence interval was determined at different TG strata.

Results: BAP between dLDL-C and cLDL across TG levels also showed unacceptable agreement at higher TG levels. Mean bias between cLDL-C and dLDL-C is low; however, it rises to +20.2 mg/dL (-17.0 to 57.5) at 250-300 and +26.5 mg/dL (-17.7 to 70.6) above 300 mg/dL. Whereas, beyond TG values >250 mg/dL, cLDL-C shows poor sensitivity. At high TG concentrations, 58.7% of samples at 250-300 mg/dL and 58.1% above 300 mg/dL are assigned to a lower LDL-C band by the Friedewald equation than by direct measurement.

Conclusions: The Friedewald equation shows acceptable agreement with the homogeneous direct LDL-C assay used in this laboratory at lower TG concentrations and unacceptable agreement at higher concentrations. At high TG concentrations (>250 mg/dL), the Friedewald equation misassigns more than half of patients to a lower LDL-C band than the homogeneous direct measurement and puts them at risk for atherosclerosis. Therefore, we recommend estimating LDL-C using Friedewald equation when serum TG is less than 250 mg/dL; beyond these levels, LDL-C should be measured directly.

## Introduction

Plasma lipoproteins are complex plasma particles composed of a hydrophobic core of cholesteryl esters and triglycerides (TGs), encased by a surface layer of free cholesterol, phospholipids, and apolipoproteins [1]. Based on their size, structure, and apolipoprotein content, the major classes of lipoproteins are chylomicrons, very-low-density lipoprotein (VLDL), low-density lipoprotein (LDL), and high-density lipoprotein (HDL) [2,3]. LDL plays a crucial physiological role by transporting cholesterol from the liver to peripheral tissues, where it is utilized for membrane synthesis, steroid hormone production, and other cellular functions. However, elevated serum LDL cholesterol (LDL-C) is recognized as a key indicator of atherosclerosis and a major risk factor for the development of cardiovascular disease [4]. The National Cholesterol Education Program Adult Treatment Panel III has recommended serum LDL-C as the primary therapeutic target in dyslipidemia management, emphasizing its central role in cardiovascular risk reduction [5]. Although directly measured LDL-C (dLDL-C) provides greater accuracy, in routine clinical practice, LDL-C is most commonly estimated using the widely accepted Friedewald equation [6].

The reference (gold standard) method for LDL-C determination is β-quantification [7], which involves ultracentrifugation or a biphasic procedure; however, it is time-consuming, relatively costly, and requires a larger serum volume, limiting its routine clinical applicability. To address this, direct homogeneous LDL-C assays have been developed for convenient integration with automated analyzer platforms. However, the estimation of dLDL-C using the homogeneous method is associated with elevated cost and remains inaccessible in numerous laboratory settings, an issue especially pronounced in resource-limited and middle-income regions. For over four decades, clinical biochemistry laboratories have routinely estimated LDL-C using the Friedewald equation, deriving calculated LDL-C (cLDL-C) from measured values of total cholesterol (TC), HDL cholesterol (HDL-C), and TGs [8]. LDL-C can be estimated by subtracting HDL-C and VLDL cholesterol (VLDL-C) from TC, where VLDL-C is determined by dividing TG by a factor of 5 [8]. The Friedewald equation is considered to be valid for specimens where TG is less than 400 mg/dL.

Most clinical laboratories estimate serum LDL-C levels from the Friedewald equation by employing serum TG, TC, and HDL-C levels. It is based on the assumption that TG is mainly present in chylomicrons and VLDL, assuming non-HDL-C (TC-HDL-C) has little effect. However, when TG levels are too high, LDL-C values tend to be underestimated. This typically occurs in the postprandial state or in patients who have normal non-HDL-C levels but elevated TG concentrations. However, the use of this constant ratio has been questioned, as VLDL represents a heterogeneous group of lipoproteins with variable TG-to-cholesterol proportions, which may lead to inaccuracies in LDL-cholesterol estimation. Several studies have assessed the impact of TG on the Friedewald equation. These studies suggest LDL may be overestimated or underestimated by the Friedewald equation at different TG levels [9-11].

The Friedewald equation was developed on the assumption that minor inaccuracies in VLDL-C estimation would have minimal impact, as VLDL-C constitutes a relatively small fraction compared with LDL-C. However, this premise warrants reconsideration in the current therapeutic landscape, where achieving very low LDL-C levels is a key clinical goal. Moreover, the growing burden of hypertriglyceridemia in the Indian subcontinent, driven by increasing prevalence of obesity, insulin resistance, and diabetes mellitus, further challenges the validity of this assumption and may compromise the accuracy of LDL-C estimation. South Asians have a unique lipid profile and are characterized by high fasting and postprandial TG with smaller dense LDL particles, which may predispose them to premature coronary heart disease [12,13]. The Friedewald equation tends to underestimate LDL-C, especially if TG levels are ≥150 mg/dL [14]. Both overestimation and underestimation can pose issues; an overestimation of LDL-C can result in unnecessary medication prescriptions, while underestimation can delay appropriate treatment. A few studies from India have reported discordant conclusions on this topic [15-18].

The aim of this study was to evaluate the agreement between cLDL-C using the Friedewald equation and dLDL-C for dyslipidemia classification across TG strata. We further quantified diagnostic misclassification rates and assessed the sensitivity and accuracy of the Friedewald equation within each TG stratum.

## Materials and methods

The study protocol was approved by the Institutional Human Ethics Committee of All India Institute of Medical Sciences (AIIMS), Bhopal (IHEC/LOP/2021/IM0384), and a waiver of informed written consent was obtained. The study population and design were based on a hospital-based cross-sectional ambispective study. Data were collected between 2021 and 2022. The sample size was determined a priori using G*Power software (Heinrich-Heine-Universität Düsseldorf, Düsseldorf, Germany). With a type I error of 0.01, a type II error of 0.01 (corresponding to a statistical power of 99%), and an anticipated Pearson correlation coefficient of 0.1 between dLDL-C and cLDL-C, the required sample size was estimated to be 1,317. A total of 1,387 samples were ultimately included in the study. Randomly selected patient samples, regardless of clinical condition, received at the Clinical Biochemistry Laboratory of AIIMS Bhopal for lipid profile testing were considered for inclusion.

Measurement of cholesterol and TG

Lipid profile (TC, TG, and HDL-C) was analyzed using a fully automated clinical chemistry analyzer (Beckman Colter India Pvt. Ltd., Mumbai, India) as standard practice, with cLDL-C calculated using the Friedewald equation [7]. dLDL-C was measured using a homogeneous method (Beckman Colter) on a random-access, fully automated clinical chemistry analyzer (Beckman Colter). In homogeneous assays, LDL-C is selectively isolated from other lipoprotein fractions using a specific combination of surfactants, allowing its direct quantification on an automated chemistry analyzer. Briefly, the homogeneous assay consists of two distinct phases. In the first phase, a unique detergent mixture solubilizes cholesterol from non-LDL lipoproteins, and the cholesterol is oxidized by cholesterol esterase, cholesterol oxidase, and peroxidase to generate a colorless product. In the second phase, another detergent mixture solubilizes LDL lipoproteins and releases cholesterol. Now, this cholesterol reacts with cholesterol esterase, cholesterol oxidase, and a substrate to yield a blue-colored complex, which is measured at 540/660 nm. This homogeneous method shows linearity in the range of 7.0 mg/dL to 400.0 mg/dL of LDL-C. Within-run and between-run precision is less than 1% and 2.5% coefficient of variation, respectively [19].

Data stratification

We used dLDL-C estimated using the homogeneous method as the true value across different TG concentration ranges. Stratified analysis was performed to assess correct classification and potential misclassification (under- and overestimation) of cLDL-C values vs. dLDL-C. The TG strata follow 50 mg/dL increments across the range over which the Friedewald equation is applied in routine practice, with boundaries aligned to values in common clinical use (150 mg/dL, the threshold for hypertriglyceridemia; 200 mg/dL; and 400 mg/dL, above which the equation is not recommended). We also compared methods when samples were stratified by TG levels (≤100, 100-150, 150-200, 200-250, 250-300, and >300 mg/dL).

Statistical analysis

Data entry was performed in Microsoft Excel 2010 (Microsoft Corporation, Redmond, WA), and then the data sheet was exported and analyzed using R (R Foundation for Statistical Computing, Vienna, Austria). Continuous variables were summarized as mean and standard deviation (SD). For the assessment between dLDL-C and cLDL-C, we categorized patients’ data into six different groups as per their level of TG: ≤100, 100-150, 150-200, 200-250, 250-300, and >300 mg/dL, at increments of 50 mg/dL. Bland-Altman plots (BAPs) were used to analyze the degree of agreement between dLDL-C and cLDL-C. BAP provides a graphical comparison of the level of agreement between the two methods, which was assessed by calculating the bias, expressed as the mean difference along with its SD. Mean bias and SD with 95% confidence interval (CI) were calculated for BAP. Scatterplots of cLDL-C vs. dLDL-C at different TG strata were plotted. We used the Pearson correlation coefficient and Lind’s concordance correlation coefficient (LCCC) to evaluate the correlation between SV and CO between the two methods (dLDL-C and cLDL-C). Sensitivity, specificity, PPV, NPV, accuracy, and kappa with 95% CI of the Friedewald equation for determining c-LDL-C were analyzed.

## Results

A total of 1,387 samples were included in this study. Data from these samples were visualized and explored for any extreme or implausible values and missing observations. Then, the dataset was filtered, and available data from 1,368 samples were considered for further analysis (see the Appendix showing results of exploratory data analysis). However, of these 1,368 samples, 80 samples had TG levels higher than 400 mg/dL. These cases were not included in the LDL calculation using Friedewald equation. Thus, the final analysis included 1,288 samples. The distribution of lipid parameters is shown in Table 1.

**Table 1:** Distribution of lipid parameters HDL-C: high-density lipoprotein cholesterol; LDL-C: low-density lipoprotein cholesterol; VLDL-C: very low-density lipoprotein cholesterol; SD: standard deviation; IQR: interquartile range

Characteristic	Value (N = 1,288)	
	Mean (SD)	Median (IQR)
Total cholesterol (mg/dL)	164.6 (50.1)	161.4 (131.9-192.9)
Triglycerides (mg/dL)	156.4 (86.7)	130.0 (88.9-217.7)
HDL-C (mg/dL)	42.4 (12.2)	41.8 (35.0-49.7)
LDL-C calculated (mg/dL)	90.9 (37.2)	89.4 (65.0-112.4)
VLDL-C (mg/dL)	31.2 (17.2)	26.0 (17.8-43.4)
LDL-C direct (mg/dL)	90.8 (38.4)	87.0 (64.7-110.9)

dLDL-C vis-a-vis calculated

Our objective was to assess the reliability of the cLDL-C value with the dLDL-C (comparator) value. BAPs were used to visualize the reliability of numerical measurements [20]. In these plots, the average value of the two methods is plotted on the X-axis, and the difference between the two methods for a given observation is plotted on the Y-axis. The variation in difference is visualized, and dotted horizontal lines at 1 or 2 SDs of the difference are plotted on the Y-axis. In our case, because we had a comparator method, i.e., dLDL-C, we have plotted it on the X-axis instead of plotting the average (Figure 1A). Figure 1A shows fair reliability between the two measures up to an LDL-C level of 150, and then many observations can be seen outside the acceptable line. Another metric used to estimate reliability is LCCC, and it is 0.89, which can be considered overall good agreement (Figure 1A) [21]. Then, we regressed cLDL-C on dLDL-C, and the parameters of the linear regression are shown on the scatterplot (Figure 1B).

**Figure 1:**
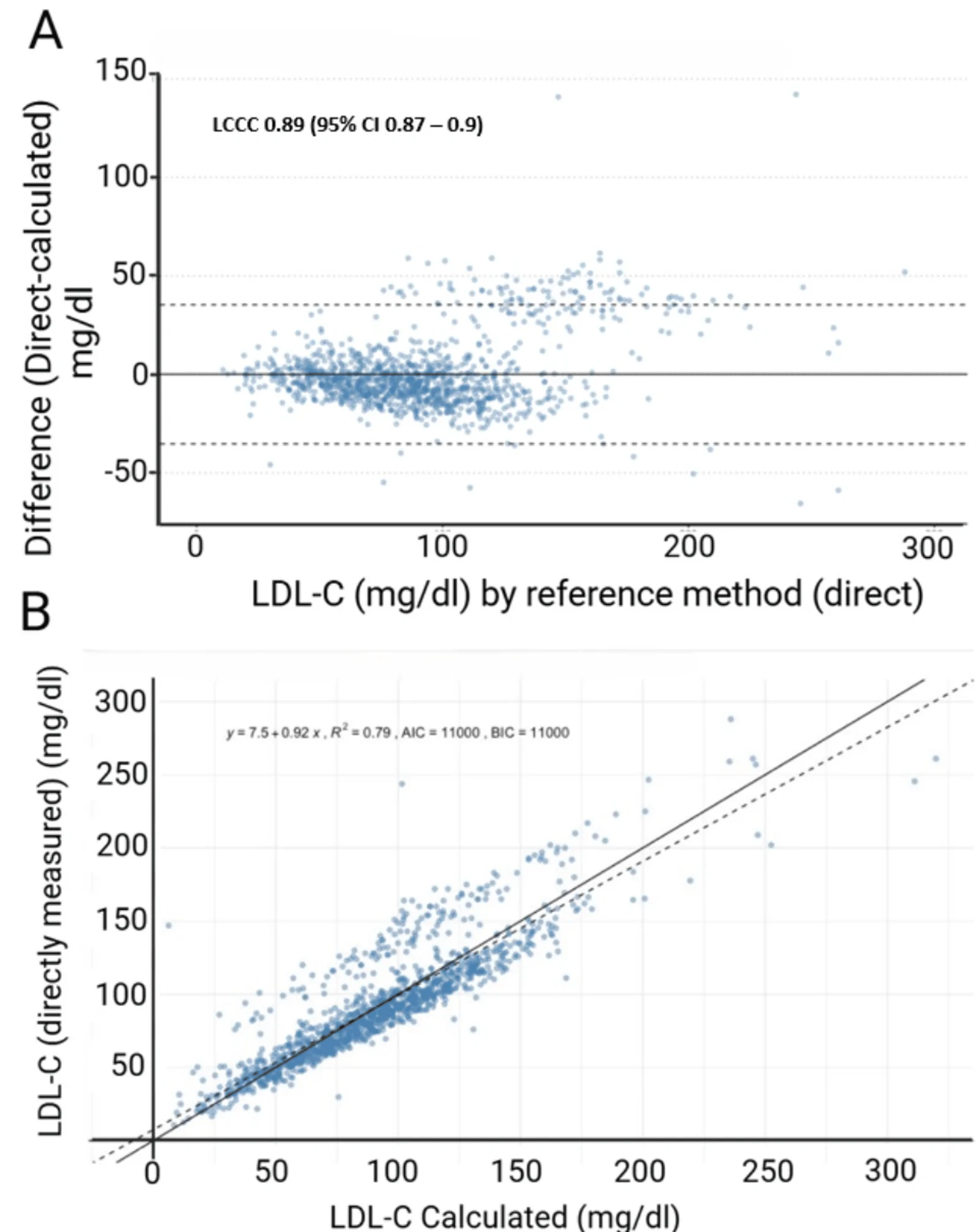
(A) Bland-Altman plot for LDL by direct vs. calculated estimate for overall data. (B) Scatterplot showing LDL by direct methods vs. calculated for overall data. LDL-C (directly measured) and LDL-C (calculated) are presented in mg/dL LDL: low-density lipoprotein; LDL-C: low-density lipoprotein cholesterol; CI: confidence interval

Treating the difference between the two methods as a continuous function of TG concentration, segmented (broken-stick) regression identifies a change-point at 166 mg/dL (95% CI: 147-183 mg/dL). The slope of the difference is 0.025 mg/dL per mg/dL of TG below the change-point and 0.198 mg/dL per mg/dL above it. The mean bias crosses zero at approximately 157 mg/dL. Furthermore, the deterioration is continuous and progressive, and it becomes clinically relevant >250 mg/dL of TG concentration (Figure 2).

**Figure 2:**
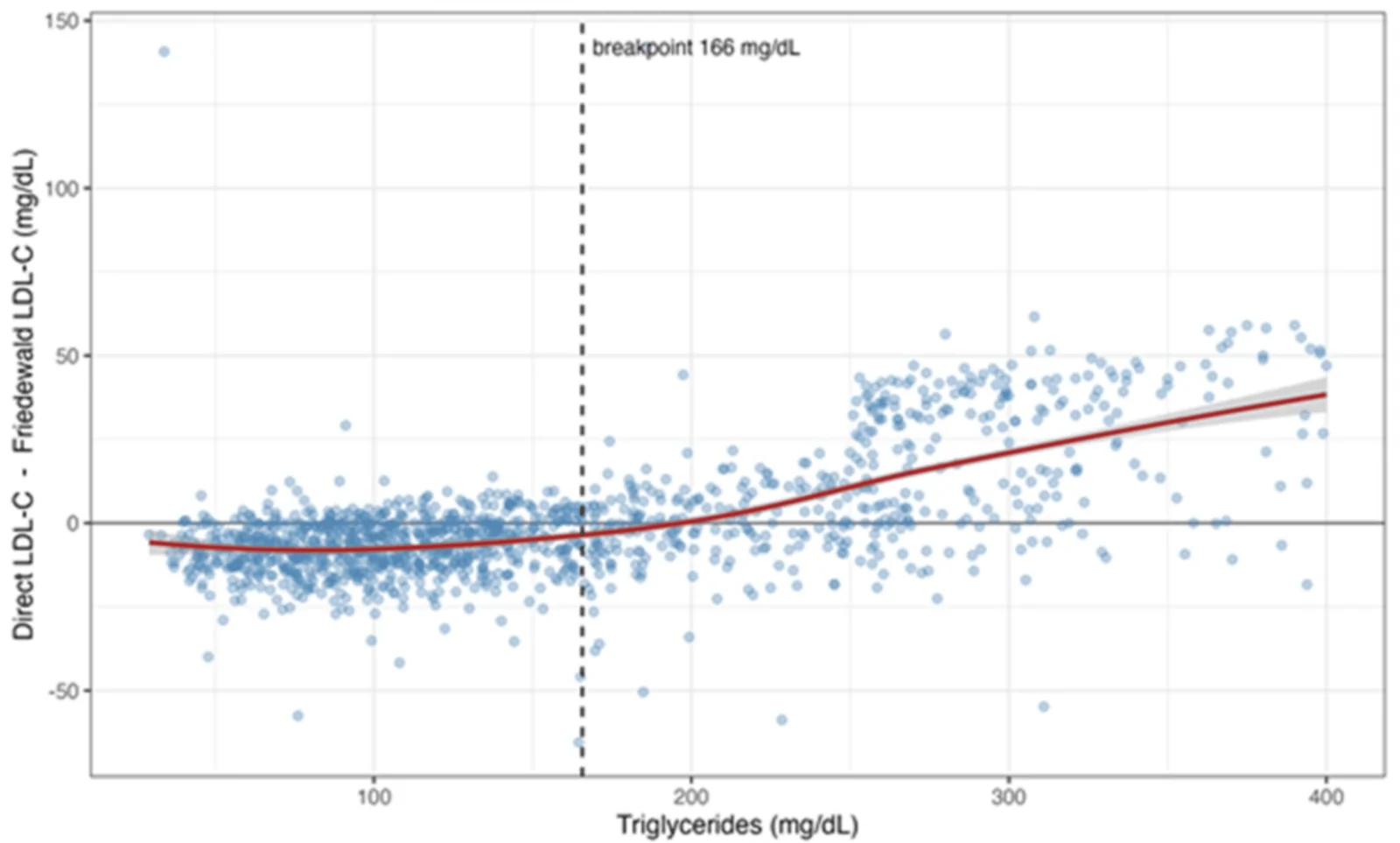
Bias in Friedewald equation increases continuously with triglycerides Each point is one sample (n = 1,288). The red line is an LOESS smoother with its 95% confidence band. The dashed vertical line marks the change point estimated by segmented regression: 166 mg/dL (95% CI: 147-183). The slope of the difference is 0.025 mg/dL per mg/dL of triglyceride below the change point and 0.198 above it; the mean bias crosses zero at approximately 157 mg/dL. Bias increases continuously, with no discontinuity at any category boundary LOESS: LOcally Estimated Scatterplot Smoothing; CI: confidence interval

LDL by direct vs. calculated across TG level categories

Patient data were categorized into six different groups as per their level of TG: ≤100, 100-150, 150-200, 200-250, 250-300, and >300 mg/dL. Figure 3 shows values of Pearson’s correlation coefficient (r), LCCC, and R^2^ for linear regression for a given TG level category. As TG levels increase, cLDL-C becomes less precise (Figure 4). LCCC, r, and R^2^ show high precision for cLDL-C up to the TG cutoff level of 250 mg/dL. Whereas beyond TG values >250 mg/dl, cLDL-C shows poor precision as LCCC, r, and R^2^ decrease sharply (Figures 3, 4).

**Figure 3:**
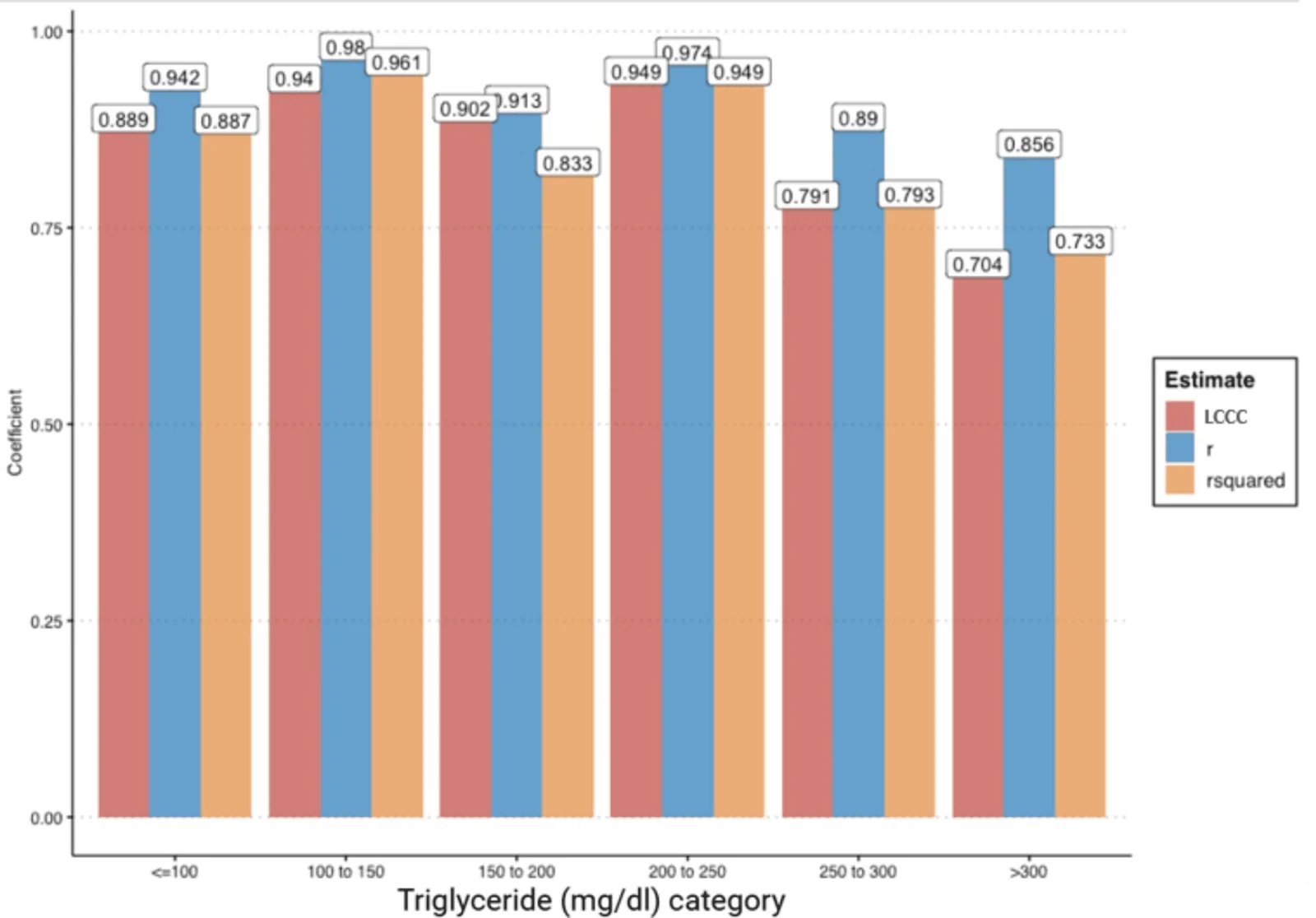
Pearson correlation coefficient (r), Lind’s concordance correlation coefficient, and R-squared for linear regression for different triglyceride categories. Triglyceride values are in mg/dL

**Figure 4:**
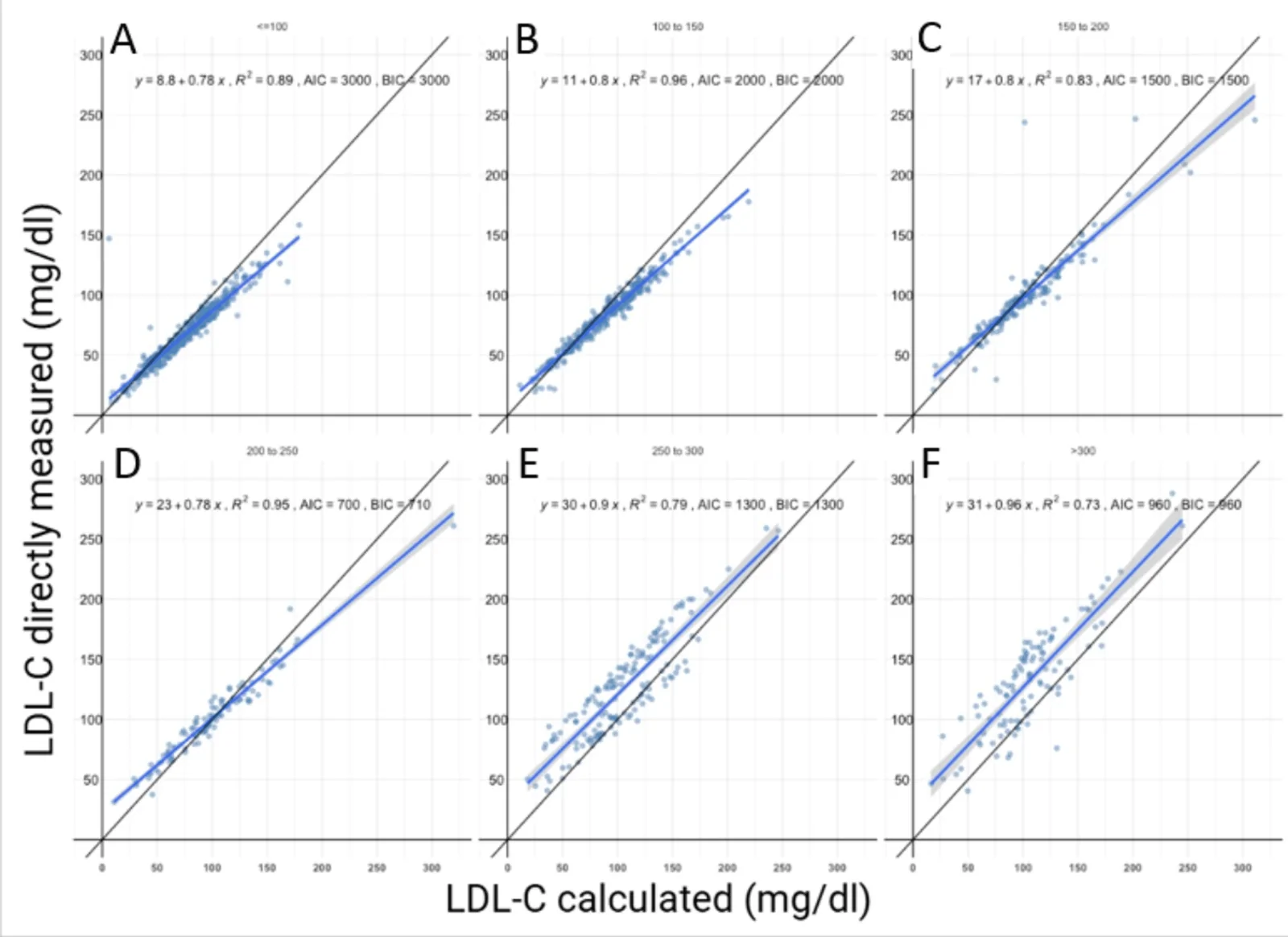
(A-F) Scatterplots of LDL-C (calculated) vs. LDL-C (direct measurement) at different TG categories. Values are in presented in mg/dL LDL-C: low-density lipoprotein cholesterol; TG: triglyceride; AIC: Akaike information criterion; BIC: Bayesian information criterion

Prediction of LDL from cLDL across TG levels

Figure 4 displays scatterplots of LDL estimated by direct measurement vs. calculated using the equation within each category of TG levels. It can be seen that R^2^ is lower for high TG levels (>250 mg/dL). BAPs between dLDL-C and cLDL-C across TG levels also show less reliability at higher TG levels, particularly for the category of TG >250 mg/dL (Figure 5).

**Figure 5:**
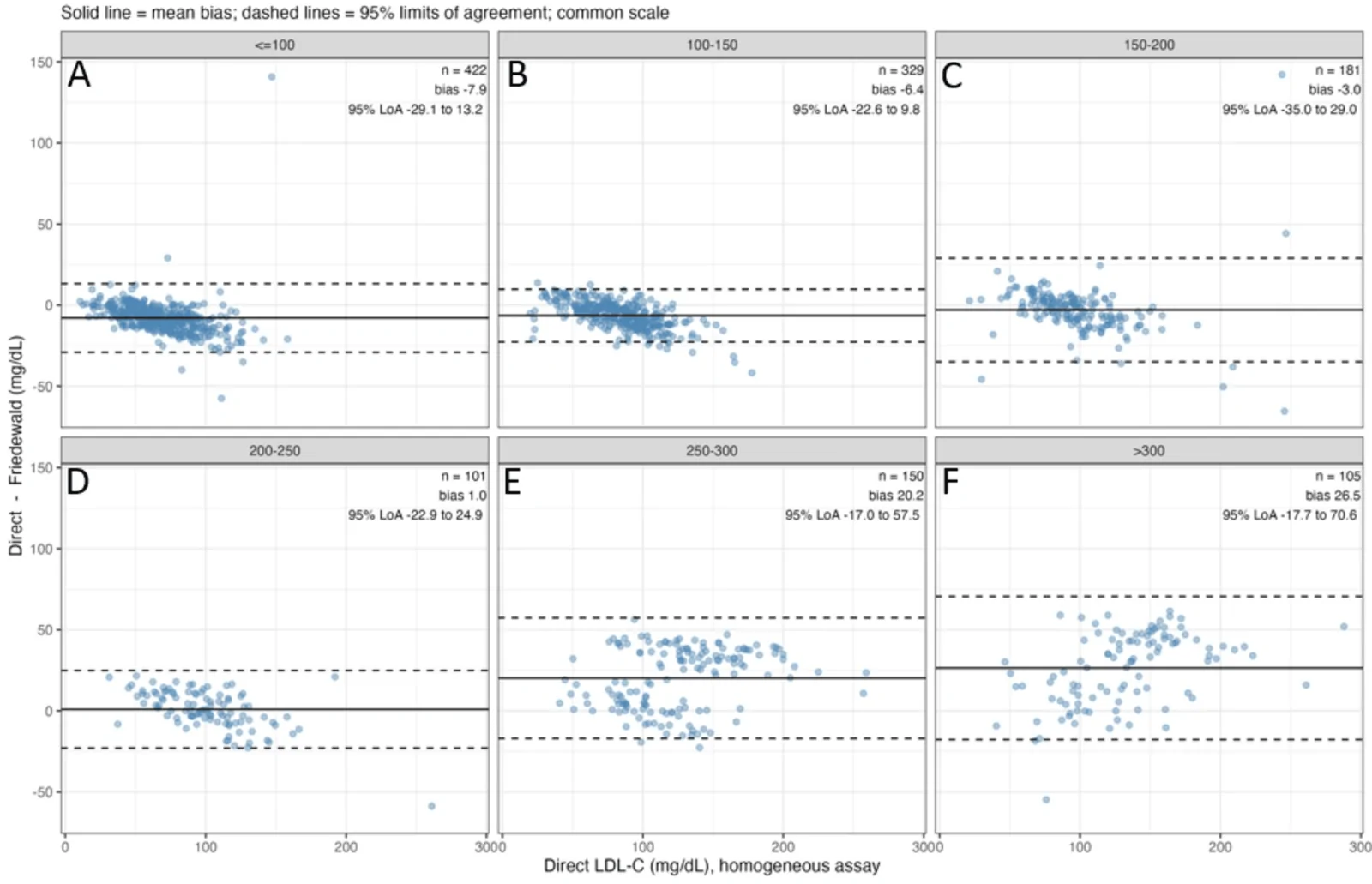
(A-F) Bland-Altman plots by triglyceride category LoA: limits of agreement; LDL-C: low-density lipoprotein cholesterol

We then calculated mean bias between the cLDL-C (Friedewald Equation) and dLDL-C for each TG stratum. Table 2 shows the number of samples, mean bias with 95% CI, SD of the bias, 95% limits of agreement, mean percentage bias, Pearson's r, and LCCC with 95% CI. Bias is defined throughout as dLDL-C minus cLDL-C, so that a positive value denotes underestimation by the Friedewald equation. As shown in Table 2 and Figure 5, the mean bias is -7.9 mg/dL (95% LoA: -29.1 to 13.2) at TG ≤100 mg/dL, -6.4 mg/dL (-22.6 to 9.8) at 100-150, -3.0 mg/dL (-35.0 to 29.0) at 150-200, and +1.0 mg/dL (-22.9 to 24.9) at 200-250. It rises to +20.2 mg/dL (-17.0 to 57.5) at 250-300 and +26.5 mg/dL (-17.7 to 70.6) above 300 mg/dL. The limits of agreement widen substantially in the two highest strata, which is the finding of clinical importance. As shown in Table 2 and Figure 5, the Friedewald equation performs worse at high TG concentrations than originally reported. Mean bias at 95% CI at different TG strata is shown in Figure 6.

**Table 2:** Bias, 95% limits of agreement, and concordance between calculated and direct LDL-C, by TG stratum Bias = direct LDL-C - calculated LDL-C, in mg/dL; positive values indicate underestimation by the Friedewald equation. Note that the direction of bias reverses between the lower and upper strata, and that the limits of agreement widen markedly above 250 mg/dL TG: triglyceride; CI: confidence interval; SD: standard deviation; LCCC, Lind's concordance correlation coefficient; LDL-C: high-density lipoprotein-cholesterol

TG stratum (mg/dL)	n	Mean bias (95% CI)	SD of bias	95% limits of agreement	Mean % bias	Pearson r	LCCC (9 5% CI)
All (TG ≤400)	1,288	-0.07 (-1.05 to 0.92)	18.02	-35.4 to 35.3	-1.8	0.89	0.89 (0.87-0.90)
≤100	422	-7.94 (-8.98 to -6.91)	10.80	-29.1 to 13.2	-10.8	0.94	0.89 (0.87-0.91)
100-150	329	-6.42 (-7.32 to -5.53)	8.27	-22.6 to 9.8	-6.4	0.98	0.94 (0.93-0.95)
150-200	181	-2.97 (-5.37 to -0.58)	16.33	-35.0 to 29.0	-2.5	0.91	0.90 (0.87-0.92)
200-250	101	1.03 (-1.38 to 3.44)	12.20	-22.9 to 24.9	3.7	0.97	0.95 (0.93-0.96)
250-300	150	20.24 (17.18 to 23.30)	18.99	-17.0 to 57.5	16.6	0.89	0.79 (0.73-0.84)
>300	105	26.47 (22.11 to 30.83)	22.53	-17.7 to 70.6	19.0	0.86	0.70 (0.62-0.77)

**Figure 6:**
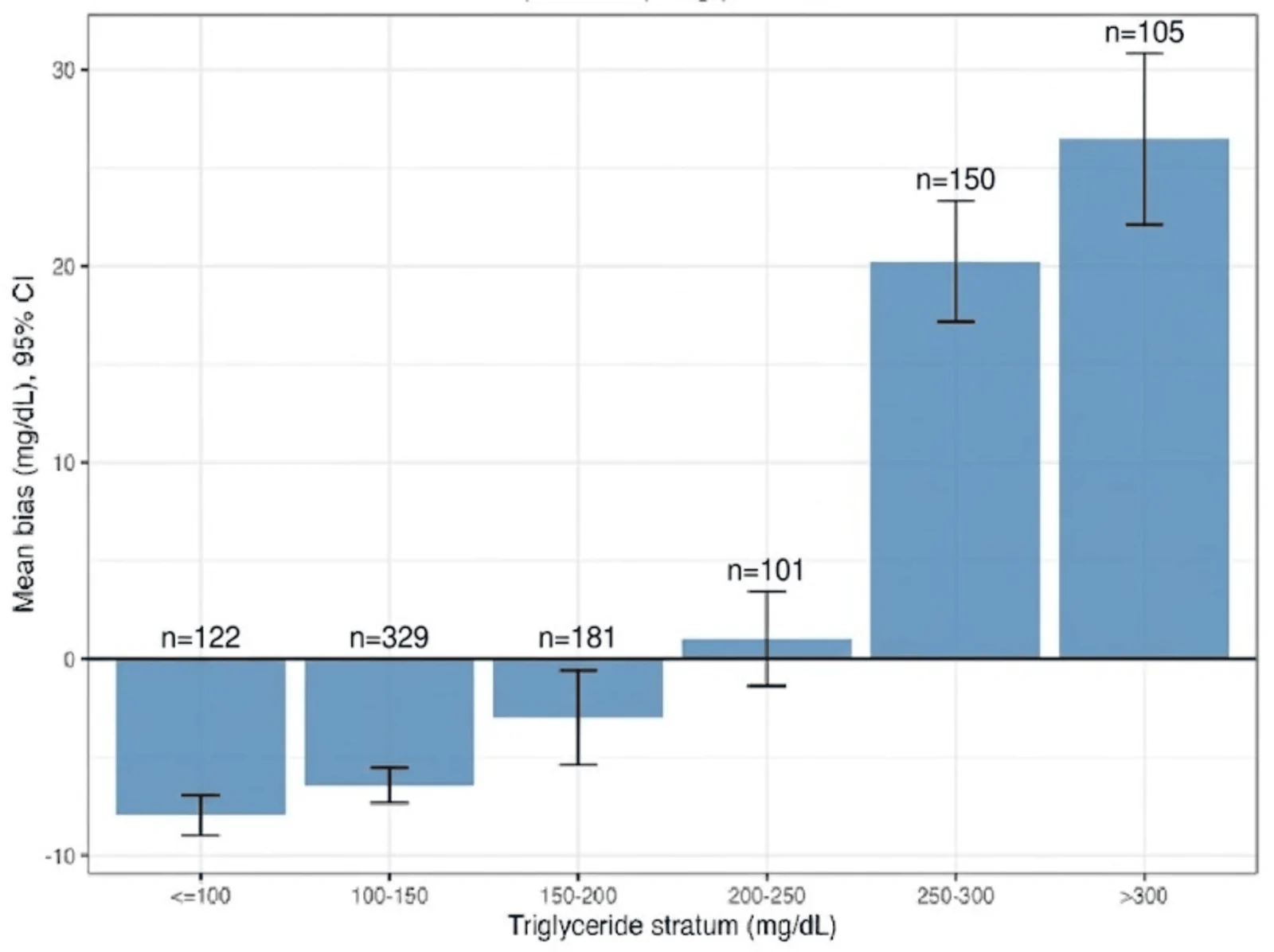
Mean bias of the Friedewald equation by triglyceride stratum, with 95% confidence intervals Error bars are 95% confidence intervals for the mean bias; the number of samples is shown above each bar. Bias is negative (overestimation by the Friedewald equation) below approximately 157 mg/dL and positive (underestimation) above it CI: confidence interval

cLDL-C misclassifies patients with higher TG levels

According to the Adult Treatment Panel III (ATP III), LDL-C <100 mg/dL is considered an optimal value, whereas LDL-C between 100 and 129 mg/dL is considered near-optimal. We categorized patients into six groups according to TG values. We then assessed whether the cLDL-C could diagnose dyslipidemia patients accurately at different TG values. Considering the 100 mg/dL cutoff value of dLDL-C, as shown in Table 3, cLDL-C was able to classify dyslipidemia patients with an accuracy of 91.8%-97.8% with TG values in the range of <250 mg/dL. However, sensitivity decreased drastically to 70.4% and 70.9% at TG values of 250-300 and >300 mg/dL, respectively. Similarly, using the 130 mg/dl cutoff for dLDL-C (Table 4), cLDL-C classified dyslipidemia patients with a sensitivity of 93.3-100% at TG values of 150-250 mg/dL. However, the sensitivity decreased drastically to 59.4% and 32.7% at TG values of 250-300 and >300 mg/dL, respectively. In addition, the sensitivity of c-LDL-C was lower (75%) in subjects with lower TG concentration (<100 mg/dL).

**Table 3:** Classification performance of calculated LDL-C against direct LDL-C at the 100 mg/dL threshold, by triglyceride stratum A sample is classified as elevated when LDL-C ≥100 mg/dL. Direct LDL-C is the comparative classification. 95% confidence intervals are exact binomial PPV: positive predictive value; NPV: negative predictive value; TG: triglyceride; CI: confidence interval; LDL-C: high-density lipoprotein cholesterol

TG stratum (mg/dL)	n	Sensitivity % (95% CI)	Specificity % (95% CI)	PPV % (95% CI)	NPV % (95% CI)	Accuracy % (95% CI)	κ
All (TG ≤400)	1,288	85.7 (82.1-88.8)	88.5 (86.2-90.6)	79.6 (75.7-83.1)	92.3 (90.2-94.0)	87.6 (85.7-89.3)	0.73
≤100	422	97.8 (88.2-99.9)	88.6 (84.9-91.6)	50.6 (39.6-61.5)	99.7 (98.3-100)	89.6 (86.3-92.3)	0.61
100-150	329	98.9 (93.8-100)	85.5 (80.5-89.7)	71.1 (62.1-79.0)	99.5 (97.4-100)	89.1 (85.2-92.2)	0.75
150-200	181	97.3 (90.5-99.7)	88.0 (80.3-93.4)	84.5 (75.0-91.5)	97.9 (92.7-99.7)	91.7 (86.7-95.3)	0.83
200-250	101	91.8 (80.4-97.7)	96.2 (86.8-99.5)	95.7 (85.5-99.5)	92.6 (82.1-97.9)	94.1 (87.5-97.8)	0.88
250-300	150	70.4 (60.8-78.8)	95.2 (83.8-99.4)	97.4 (91.0-99.7)	55.6 (43.4-67.3)	77.3 (69.8-83.8)	0.54
>300	105	70.9 (59.6-80.6)	92.3 (74.9-99.1)	96.6 (88.1-99.6)	51.1 (36.1-65.9)	76.2 (66.9-84.0)	0.50

**Table 4:** Classification performance of calculated LDL-C against direct LDL-C at the 130 mg/dL threshold, by TG stratum A sample is classified as elevated when LDL-C ≥130 mg/dL. In the highest TG stratum, the Friedewald equation identifies only one-third of samples that are ≥130 mg/dL by direct measurement PPV: positive predictive value; NPV: negative predictive value; TG: triglyceride; CI: confidence interval; LDL-C: high-density lipoprotein-cholesterol

TG stratum (mg/dL)	n	Sensitivity % (95% CI)	Specificity % (95% CI)	PPV % (95% CI)	NPV % (95% CI)	Accuracy % (95% CI)	κ
All (TG ≤400)	1,288	61.2 (53.4-68.5)	94.3 (92.7-95.6)	61.9 (54.1-69.3)	94.1 (92.6-95.4)	89.9 (88.1-91.5)	0.56
≤100	422	75.0 (19.4-99.4)	95.7 (93.3-97.4)	14.3 (3.0-36.3)	99.8 (98.6-100)	95.5 (93.1-97.3)	0.23
100-150	329	100 (75.3-100)	94.3 (91.1-96.6)	41.9 (24.5-60.9)	100 (98.8-100)	94.5 (91.5-96.7)	0.57
150-200	181	94.7 (74.0-99.9)	89.5 (83.7-93.8)	51.4 (34.0-68.6)	99.3 (96.2-100)	90.1 (84.7-94.0)	0.61
200-250	101	93.3 (68.1-99.8)	93.0 (85.4-97.4)	70.0 (45.7-88.1)	98.8 (93.3-100)	93.1 (86.2-97.2)	0.76
250-300	150	59.4 (46.4-71.5)	97.7 (91.9-99.7)	95.0 (83.1-99.4)	76.4 (67.3-83.9)	81.3 (74.2-87.2)	0.60
>300	105	32.7 (20.7-46.7)	94.0 (83.5-98.7)	85.7 (63.7-97.0)	56.0 (44.7-66.8)	61.9 (51.9-71.2)	0.26

Assigning patients to a lower LDL-C category by calculation is clinically hazardous because it may lead to withheld or underintensified treatment. We estimated what percentage of patients are assigned to a lower category of LDL-C by the Friedewald equation. As shown in Table 5, 58% of patients are misassigned to a lower category by cLDL-C, which is a clinically significant proportion. The proportion of samples assigned to a different band by the two methods rises from 25.5% at TG 100-150 mg/dL to 62.7% at 250-300 and 63.8% above 300. More importantly, the direction of misassignment reverses. At low TG concentrations, the discordance is almost entirely upward (35.3% assigned to a higher band at TG ≤100), which would at worst prompt unnecessary intensification. At high TG concentrations, it is almost entirely downward: 58.7% of samples at 250-300 mg/dL and 58.1% above 300 mg/dL are assigned to a lower LDL-C band by the Friedewald equation than by direct measurement, which is the clinically hazardous direction because it may lead to treatment being withheld or underintensified.

**Table 5:** Reclassification across guideline LDL-C categories, by triglyceride stratum Categories are <55, 55-69, 70-99, 100-129, 130-159, and ≥160 mg/dL TG: triglyceride; LDL-C: high-density lipoprotein cholesterol

TG stratum (mg/dL)	n	Same category %	Different category %	Calculated assigns a lower category, %	Calculated assigns a higher category, %
≤100	422	63.3	36.7	1.4	35.3
100-150	329	74.5	25.5	2.7	22.8
150-200	181	74.0	26.0	6.1	19.9
200-250	101	71.3	28.7	17.8	10.9
250-300	150	37.3	62.7	58.7	4.0
>300	105	36.2	63.8	58.1	5.7

We then conducted an exploratory, hypothesis-generating analysis to estimate whether an alternative equation at higher TG performs better. We calculated VLDL by dividing TG by 6 in patients with TG concentrations >250 mg/dL. As shown in Table 6, mean bias decreased to 11.18 and 16.89 at TG concentrations of 250-300 and >300 mg/dL. This study was not designed to compare LDL-C estimating equations, and no formal comparison against a reference measurement procedure was possible. Thus, the alternate equation performs better at higher TG concentrations (>250 mg/dL).

**Table 6:** Exploratory comparison with a modified VLDL-C divisor TG: triglyceride; VLDL-C: very high-density lipoprotein cholesterol

TG stratum (mg/dL)	n	Mean bias, Friedewald (TG/5)	Mean bias, TG/6 above 250 mg/dL
≤100	422	-7.94	-7.96
100-150	329	-6.42	-6.59
150-200	181	-2.97	-3.02
200-250	101	1.03	0.76
250-300	150	20.24	11.18
>300	105	26.47	16.89

## Discussion

The Friedewald equation, introduced in 1972, gained widespread acceptance in clinical practice as a cost-effective and convenient alternative to ultracentrifugation for dLDL-C measurement [8]. Despite its continued routine use, the equation is based on the assumption that VLDL-C can be approximated as TG divided by a fixed factor of 5 (TG/5). This simplification, however, does not consistently yield accurate estimates, as VLDL comprises a heterogeneous population of lipoproteins with variable TG-to-cholesterol ratios [22]. Consequently, reliance on a fixed divisor may introduce systematic errors in LDL-C estimation. Importantly, these inaccuracies become more pronounced under conditions where precise LDL-C estimation is critical, particularly in patients with elevated TG levels. In this context, our study was designed to evaluate the performance of Friedewald equation across different TG strata. We specifically examined cases with TG levels ≥250 mg/dL, where LDL-C underestimation is more likely and may lead to clinically significant misclassification, especially relative to high-risk therapeutic targets such as LDL-C <100 mg/dL. Our study demonstrates that at TG concentrations (>250 mg/dL), c-LDL-C estimated using the Friedewald equation misclassifies 58% of patients in the lower LDL-C category. The mean bias is low at low-to-moderate concentrations (TG ≤100 to 250 mg/dL), whereas it is high at >250-300 mg/dL. The mean bias of -7.9 mg/dL at TG below 100 mg/dL, although precisely estimated and statistically distinguishable from zero, is unlikely to change management for most patients, whereas the mean bias of +20.2 mg/dL at 250-300 mg/dL is of a magnitude that routinely crosses treatment thresholds and can lead to misclassification of more than half of the patients to a lower category, putting them at risk for atherosclerosis. Further, the two highest strata of TG together contribute 255 samples (19.8%), and estimates within them are correspondingly less precise, which is why CIs are now given for every estimate.

Several studies across diverse populations and disease conditions have critically evaluated the accuracy of the Friedewald equation and proposed alternative approaches for more precise LDL-C estimation. A large database study of 14,620 lipid profile samples from South India found that the Friedewald equation correlated best with directly measured LDL at TG levels 100-200 mg/dL. The study showed that the median directly measured LDL was 9.0 mg/dL higher than Friedewald-estimated LDL when the estimated LDL was <70 mg/dL. At TG levels 200-399 mg/dL, the underestimation was even greater (median 18.4 mg/dL higher) [18]. In the Lipid Research Clinics Prevalence Study, DeLong et al. reported that the mean TG:VLDL-C ratio varied considerably, ranging from 5.2 to 8.9 across different clinical centers, underscoring the limitation of assuming a fixed ratio [23]. Furthermore, in patients with markedly elevated TG levels (≥400 mg/dL), accurate estimation of LDL-C necessitates β-quantification using ultracentrifugation, which remains the reference method [17]. To address these limitations, Martin et al. proposed a novel method for LDL-C estimation [LDL-C(M)], calculated as $\mathrm{TC} - \mathrm{HDL\mathrm{-}C} - \mathrm{\frac{TG}{\text{Adjustable factor}}}$, where the adjustable factor is derived from strata-specific median TG:VLDL-C ratios based on a large dataset of over 900,000 individuals in the United States [18].

The adjustable factor was determined as the strata-specific median TG:VLDL-C ratios from a dataset of 900,605 subjects in the United States, which appeared to provide a more accurate measure of LDL-C than that calculated with the Friedewald equation. This approach has been shown to improve the accuracy of LDL-C estimation compared to Friedewald equation. However, important determinants such as ethnicity, obesity, diabetes, and insulin resistance, which are particularly relevant in populations such as the Indian subcontinent, have not been adequately accounted for and may influence the TG:VLDL-C relationship. In parallel, homogeneous assays for dLDL-C, based on selective solubilization techniques, have been developed and are increasingly used in clinical laboratories. These methods have demonstrated good agreement with cLDL-C values in several studies, offering a practical alternative that circumvents the inherent assumptions of estimation formulas [16].

DeLong et al. proposed the use of a fixed factor of 6 for estimating VLDL-C, thereby recalibrating the population mean TG:VLDL-C ratio; however, this modification did not account for the substantial interindividual variability in this relationship [23-25]. It is also important to consider that both the original Friedewald equation and the DeLong modification were developed in an era when achieving LDL-C levels below 70 mg/dL was not recognized as a therapeutic goal for secondary prevention in high-risk patients [23]. Consequently, LDL-C values in this lower range were either underrepresented or absent in the datasets used to derive these equations, potentially limiting their accuracy and applicability in contemporary clinical practice [8].

cLDL-C using the Friedewald equation can produce inaccurate results, either falsely low or high, particularly in the presence of high TG levels. This limitation is especially significant in populations such as individuals with diabetes, coronary artery disease, polypharmacy, type III hyperlipoproteinemia, and older adults. Relying solely on cLDL values in such cases may delay or miss necessary interventions, including lifestyle changes or initiation of lipid-lowering therapy, thereby potentially compromising patient outcomes. Moreover, falsely elevated or imprecise LDL values may lead to inappropriate medication dosing, increasing both treatment costs and the risk of adverse effects.

This study has certain limitations that should be acknowledged. First, it was conducted at a single center, which may limit the generalizability of the findings to broader populations. Second, potential confounding factors such as dietary status, comorbidities (e.g., diabetes, metabolic syndrome), and medication use were not stratified in detail, all of which may influence lipid parameters and the TG:VLDL-C relationship. A further limitation concerns the comparative method itself. Homogeneous dLDL-C assays are not free of analytical bias. Their performance depends on the surfactant chemistry of the particular assay and on the lipoprotein composition of the specimen, and several such assays have been shown to deviate from β-quantification in specimens with abnormal lipid profiles, including hypertriglyceridemia, precisely the specimens in which the present study reports the largest disagreement. Because we did not have access to β-quantification, we cannot determine how much of the divergence observed at high TG concentrations reflects error in the Friedewald estimate and how much reflects bias in the direct assay. Our findings should therefore be read as a description of agreement between cLDL-C (measured using the Friedewald equation) and the homogeneous method, not as a measurement of the accuracy of either against a reference measurement procedure.

The future perspectives are as follows. This study was performed at a single-center, tertiary care hospital; generalization of findings will require a multicentric study. Future studies should focus on validating these findings in larger, multicentric cohorts representing diverse ethnic and clinical populations, particularly in regions with a high prevalence of dyslipidemia and metabolic disorders. There is also a need to explore population-specific or condition-specific adjustment factors for improving cLDL-C accuracy. Integration of novel estimation models, such as adjustable factor-based equations, and wider adoption of cost-effective dLDL-C assays could enhance clinical utility. Furthermore, longitudinal studies assessing the impact of LDL-C misclassification on cardiovascular outcomes would provide valuable insights into optimizing lipid management strategies in routine clinical practice. We acknowledge that the study was not designed as a comparison of estimating equations, and alternative equations, the DeLong modification, the Martin-Hopkins adjustable-factor method, the Sampson equation, and population-specific formulas may perform differently in this population. As an exploratory observation only, substituting a divisor of 6 for TG above 250 mg/dL reduces the mean bias from +20.2 to +11.2 mg/dL at 250-300 and from +26.5 to +16.9 mg/dL above 300, which suggests that a locally derived adjustable factor merits formal evaluation.

## Conclusions

The present study demonstrates that the Friedewald equation shows acceptable agreement with the homogeneous direct LDL-C assay used in this laboratory at lower TG concentrations and unacceptable agreement at higher concentrations. Mean bias between cLDL-C and dLDL-C is low; however, it rises to +20.2 mg/dL (-17.0 to 57.5) at 250-300 and +26.5 mg/dL (-17.7 to 70.6) above 300 mg/dL. The limits of agreement widen substantially in the two highest strata, which is clinically relevant. Thus, the Friedewald equation remains a reliable, cost-effective, and practical method for estimating LDL-C in patients with low-to-moderate TG levels (<250 mg/dL). The Friedewald equation tends to underestimate LDL-C in Indian patients with higher TG levels (>250 mg/dL), potentially leading to clinically relevant misclassification of dyslipidemia. In patients with higher TG levels (>250 mg/dL), the Friedewald equation may misassign 58% of patients to a lower category, suggesting that c-LDL-C using the Friedewald equation may underestimate LDL-C. Furthermore, given the increasing emphasis on stringent LDL-C targets, particularly in high-risk populations, such inaccuracies may adversely impact clinical decision-making. Therefore, while the Friedewald equation may be suitably applied in patients with lower TG levels, dLDL-C should be preferred in individuals with elevated TGs to ensure accurate risk stratification and appropriate therapeutic intervention.

## Figures and Tables

**Figure 7:**
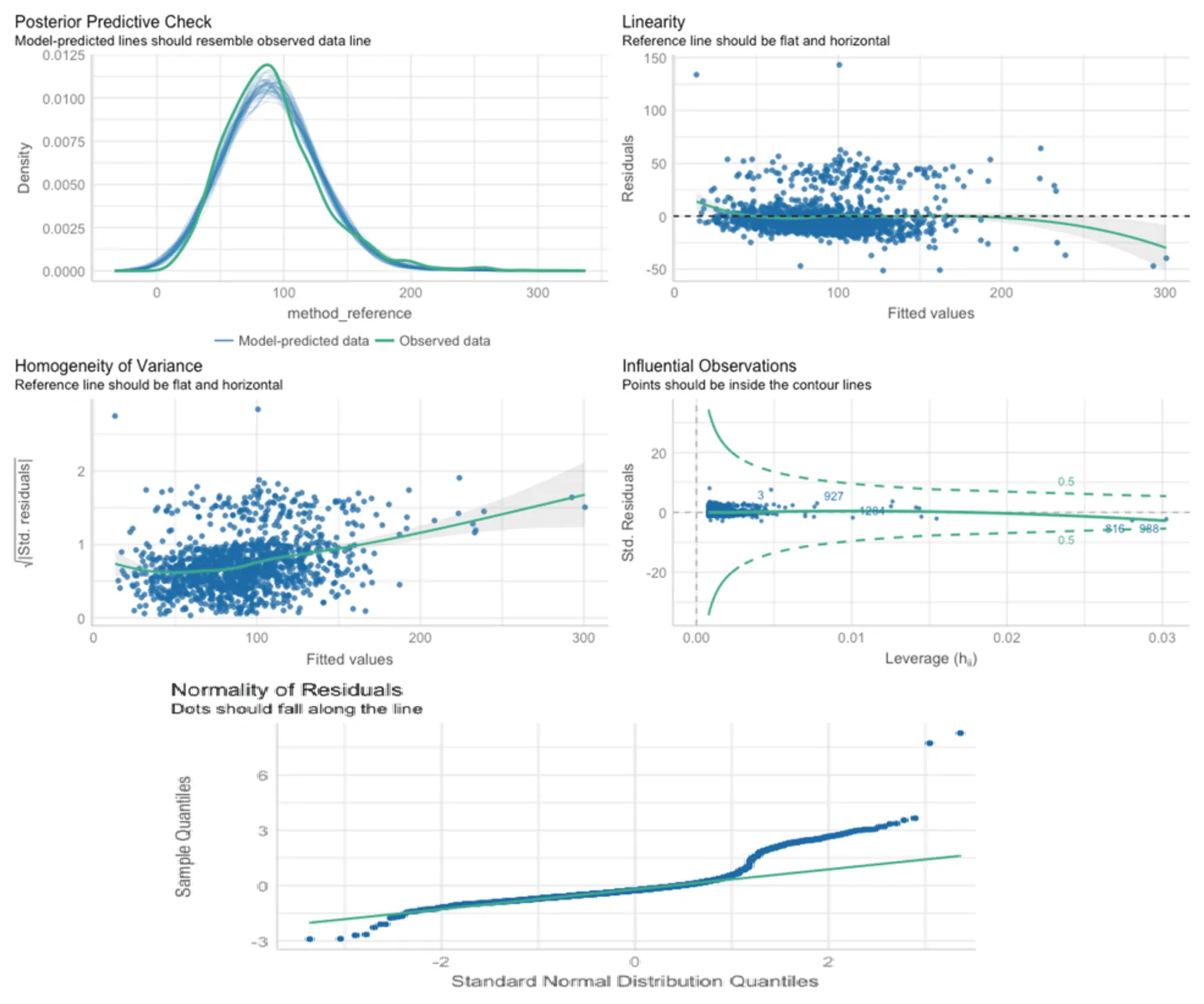
Posterior predictive check, linearity, homogeneity of variance, and influential observations and normal residuals graphs
